# Chemical Crosslinking of 6FDA-ODA and 6FDA-ODA:DABA for Improved CO_2_/CH_4_ Separation

**DOI:** 10.3390/membranes8030067

**Published:** 2018-08-20

**Authors:** Mohd Zamidi Ahmad, Henri Pelletier, Violeta Martin-Gil, Roberto Castro-Muñoz, Vlastimil Fila

**Affiliations:** 1 Department of Inorganic Technology, University of Chemistry and Technology Prague, Technická 5, 166 28 Prague 6, Czech Republic; martinv@vscht.cz (V.M.-G.); filav@vscht.cz (V.F.); 2 École Nationale Supérieure des Industries Chimique, 1 Rue Grandville—BP 20451, 54001 Nancy, France; henri.p15@live.fr

**Keywords:** gas separation, polyimide, grafting, chemical crosslinking, plasticization resistance

## Abstract

Chemical grafting or crosslinking of polyimide chains are known to be feasible approaches to increase polymer gas-pair selectivity and specific gas permeance. Different co-polyimides; 6FDA-ODA and 6FDA-ODA:DABA were synthesized using a two-step condensation method. Six different cross-linkers were used: (i) *m*-xylylene diamine; (ii) *n*-ethylamine; and (iii) *n*-butylamine, by reacting with 6FDA-ODA’s imide groups in a solid state crosslinking; while (iv) ethylene glycol monosalicylate (EGmSal); (v) ethylene glycol anhydrous (EGAn); and (vi) thermally labile iron (III) acetylacetonate (FeAc), by reacting with DABA carboxyl groups in 6FDA-ODA:DABA. The gas separation performances were evaluated by feeding an equimolar CO_2_ and CH_4_ binary mixture, at a constant feed pressure of 5 bar, at 25 °C. Fractional free volume (FFV) was calculated using Bondi’s contribution method by considering the membrane solid density property, measured by pycnometer. Other characterization techniques: thermal gravimetric analysis (TGA), differential scanning calorimetry (DSC), scanning electron microscopy (SEM), Fourier transform infrared spectroscopy (FTIR) were performed accordingly. Depending on the type of amine, the CO_2_/CH_4_ selectivity of 6FDA-ODA increased between 25 to 100% at the expense of CO_2_ permeance. We observed the similar trend for 6FDA-ODA:DABA EGmSal-crosslinked with 143% selectivity enhancement. FeAc-crosslinked membranes showed an increment in both selectivity and CO_2_ permeability by 126% and 29% respectively. Interestingly, FeAc acted as both cross-linker which reduces chain mobility; consequently improving the selectivity and as micro-pore former; thus increases the gas permeability. The separation stability was further evaluated using 25–75% CO_2_ in the feed with CH_4_ as the remaining, between 2 and 8 bar at 25 °C. We also observed no CO_2_-induced plasticization to the measured pressure with high CO_2_ content (max. 75%).

## 1. Introduction

Aromatic polyimide has become the polymer membrane of choice for natural gas separation applications due to its excellent size-sieving ability (high diffusivity selectivity) [1,2,3] to meet the required, more stringent product specifications nowadays. Moreover, their excellent mechanical properties and processability easiness attract more researchers to expand its applications further. Nevertheless, just as the other polymer types, aromatic polyimide is also restricted to CO_2_-induced plasticization and the permeability–selectivity trade-off [4]. In the presence of high CO_2_ content in the feed gas, plasticization significantly reduces the size-sieving ability, successively restricting polyimide’s usage in the industrial applications.

**Figure 1:**
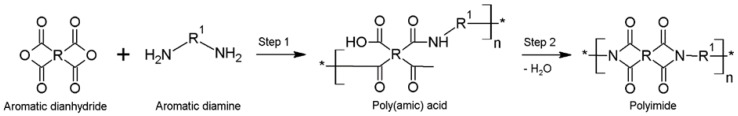
The schematic representation of a two-step synthesis method of polyimide through the formation of poly(amic) acid (PAA) and followed by an imidization process to produce polyimide. R and R^1^ are aromatic compounds.

As it has been established, with careful selection of suitable monomers (dianhydride and diamine) for polyimide syntheses the resultant chemical structure can be manipulated and optimized accordingly to the intended separation. For this study, we selected 6FDA-based aromatic polyimide (6FDA-ODA and 6FDA-ODA:DABA) for their fluorine groups (–CF_3_) in 6FDA and the bulky spatial structures of ODA which are expected to prevent chain packing compaction. As a result, polyimides with larger free volumes and higher diffusion coefficients of the permeants are produced. Moreover, the aromatic group is said to improve other polymer properties such as heat and chemical resistance, polymer chain rigidity thus giving a range of polymers with higher glass transition temperatures (T_g_ = 280–400 °C) than most common plastics [1]. Furthermore, the presence of DABA monomer could increase CO_2_ solubility due to its carboxyl group’s affinity towards the gas. Besides the mentioned intrinsic advantages, the introduction of carboxyl groups opens up ways for polyimide modifications to further improve its separation performance. These polyimides are typically synthesized through both condensation (2-steps polymerization) and addition (chain growth polymerization) methods. Condensation polymerization is a method which firstly involves a reaction between an aromatic diamine and an aromatic dianhydride in an aprotic solvent, preferably under an anhydrous condition to form a poly(amic acid) solution. An imidization process is required as the second step to obtain a polyimide, either by thermal or chemical imidization to achieve a cyclodehydration of amic acid to imide, as Figure 1 depicts [5,6,7]. Herein, the method is adapted, with thermal imidization procedure. The addition polymerization method is a simple monomer linking, which derived from the conversion of alkenes to long-chain alkanes. Additionally, this method differs from condensation polymerization as it does not co-generate other products, such as water.

Besides making polyimide into a mixed matrix membrane and benefiting from the presence of the inorganic phase in tackling plasticization [8], the procedure has proven to be more challenging when it comes to production upscaling, even more, to produce a hollow fiber membrane. Another more straightforward and less costly approach is polyimide crosslinking, which has been proven to prevent polymer swelling in the presence of plasticization agents [9,10]. However, separation performance is at the expense of gas permeability with increasing crosslinking degrees [11]; as the chain mobility is restricted, the size and the number of free volume in the polymer matrix is redistributed. Crosslinking can be carried out by either thermal treatment or chemical agents as bridges to the polymer chains [1,12].

**Figure 2:**
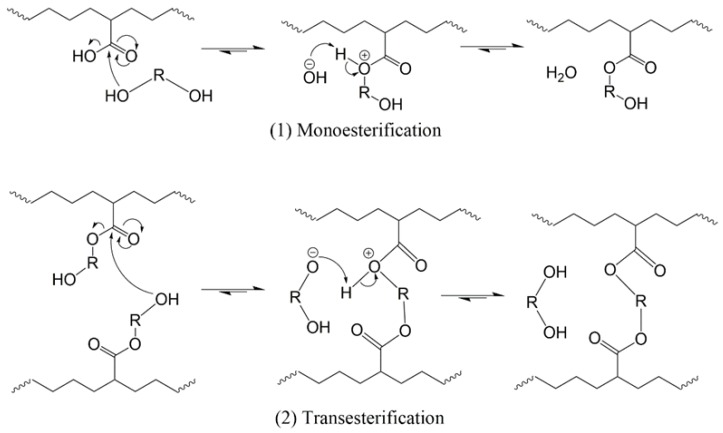
The scheme proposed for diols crosslinking with hydroxyl-containing polyimides through monoesterification and transesterification.

Polymer crosslinking is conducted to produce an assembly of linked polymer molecules. The procedure can be performed during the polymerization process or in a subsequent step after the initial formation of the polymer macromolecules [12]. In the first method, crosslinked polymers are made by a step-processes procedure, often by condensation polymerization, in the presence of monomers having group functionality greater than two [1,7,13]. The concentration of these monomers directly influences the polymer crosslinking density, and therefore the final material properties. The idea of reacting diols and carbonyl groups, thereby producing polyester has been opted onto many polyimides containing carboxyl groups [7], making diol another preferred crosslinking agent nowadays. Polyimide’s carboxyl group is first reacted with a diol in an acidic solution for a mono-esterification reaction, followed by thermal treatment to induce a trans-esterification reaction which releases half of the diols. The first step generates a diol-grafted polyimide and the second step produces diol-crosslinked polyimide. The proposed reaction is referred to in Figure 2 [14,15,16]. Another crosslinking agent that has been reported to demonstrate an excellent potential is iron (III) acetylacetonate (FeAc), an ionic thermally labile unit [17]. FeAc consists of an iron (III) ion which has high charge density to feasibly crosslink polymer chains and hinder their mobility, while the organic acetylacetonate improves the organic–inorganic compatibility and is easily removed upon thermal annealing and subsequently gives an additional free volume to the polymer matrix. Chua et al. [17] reported FeAc presented the most reproducible CO_2_ and CH_4_ separation results, compared to silver acetylacetonate, zinc acetylacetonate, and iron (III) chloride. Hence the selection of FeAc seems to be a suitable approach for our study.

**Figure 3:**
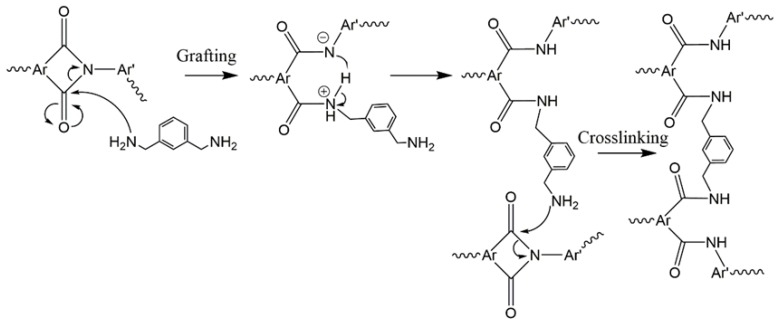
The proposed crosslinking mechanisms of polyimide using a diamine, occurs in two steps; grafting and crosslinking [13]. In the case of using single amine-functionalized compounds, only the grafting reaction occurs.

In the second method, crosslinking occurs after the formation of a solid pre-polymer, also often referred to as the ‘curing method’ or solid state crosslinking [16]. This method has been proven to improve material properties (tensile strength, strain-stress). Most importantly, in the case of polymer utilization as gas separation membrane, this method is able to modify a finished membrane to obtain desirable performances, i.e., a polymer membrane with high chain flexibility is thus highly permeable to gasses, membrane ‘curing’ is able to increase the gasses selectivity as the crosslinking reduces its chain flexibility, producing a higher packing density polymer membrane with reduced permeabilities [2,12]. The curing method has been reported in several membrane types for different separation applications, i.e., 6FDA-DAM/DABA for CO_2_/CH_4_ separation ([18]), 6FDA-NDA/DABA for H_2_/CO_2_ separation [13] and also 6FDA-NDA/DABA for ethanol dehydration via pervaporation [7]. Amine crosslinking is one of the commonly used procedures in membrane curing, and it is highly dependent on the number of amine groups and their structures, producing various crosslinking extent of a polyimide. We utilized a diamine, *m*-xylylene diamine as the curing agent, for its aromatic ring and *meta*-position amine functional groups which gives higher CO_2_/CH_4_ selectivity improvement as reported in 6FDA-2,6-DAT crosslinking using a *meta-* and *para*-position aromatic diamine [19]. Both diamines produce higher chain packing membranes than its respective un-crosslinked membrane; however, the shorter amine group distance in *meta*-position diamine may further increase chain packing, simultaneously lowering its free volume to achieve higher selectivity. The use of aliphatic amines is for performance comparison. The proposed polyimide–diamine crosslinking mechanism is referred to Figure 3.

In this study, we synthesized 6FDA-ODA and 6FDA-ODA:DABA and explored both crosslinking methods onto the polymers using several crosslinking agents: (1) step-processes polymerization, obtained through mono- and trans-esterification reaction of a polyimide using ethylene glycol monosalicylate and ethylene glycol anhydrous, also using a thermally labile unit, iron (III) acetylacetonate; (2) curing method using aromatic *m*-xylylene diamine, a large and rigid crosslinker and aliphatic single-amine compounds, *n*-ethylamine and *n*-butylamine for a comparison. The main purposes of this study are to: (1) study the effects of various cross-linking modifications on morphology and physicochemical properties of the resultant polyimide flat sheet membranes; (2) investigate the membrane performance for CO_2_/CH_4_ as a function of the crosslinking modification.

## 2. Materials and Methods

The synthesis of 6FDA-ODA and 6FDA-ODA:DABA were conducted through a classic two-step (condensation) polymerization by reacting a one-to-one stoichiometric amount of a dianhydride and a diamine in a polar aprotic solvent under the N_2_ atmosphere, to produce a 10 wt.% polymer concentration of poly(amic) acid (PAA) solution. The obtained PAA was thermally and gradually annealed between 70 and 300 °C for imidization. For the synthesis of 6FDA-ODA, 9 mmol (4.0 g) of 4,4′-(hexafluoroisopropylidene) diphthalic anhydride (6FDA, 99%, Sigma-Aldrich, St. Louis, MO, USA), 9 mmol (1.8 g) of 4,4′-oxydianiline (ODA, 97%, Sigma-Aldrich) in 58 g of *n,n*-dimethylformamide (DMF, anhydrous, ≥99.9%, Sigma-Aldrich). In the case of 6FDA-ODA:DABA (8:2 diamine molar ratio), 7.2 mmol (1.44 g) of ODA was used with 0.8 mmol (0.12 g) 3,5-diaminobenzoic acid (DABA, 98%, Sigma-Aldrich). The dianhydride was dried before the synthesis by vacuum drying at 160 °C for 6–7 h to discard moisture in the monomer, while the diamines were used as received.

The diamine crosslinking was conducted on the annealed 6FDA-ODA where the flat sheet membranes were immersed into a solution of 1 wt.% *m*-xylylene diamine (99%, Sigma-Aldrich) in methanol (MeOH, >99.8%, Penta Chemicals, Prague, Czech republic) overnight, followed by oven drying at 60–70 °C for 4–6 h. The same procedure was followed for *n*-ethylamine (anhydrous, >99.5%, Sigma-Aldrich) and *n*-butylamine (99.5%, Sigma-Aldrich) crosslinking. Crosslinking with ethylene glycol is a two-step esterification process. A ca. 1 g of ethylene glycol anhydrous (EGAn, 99.8%, Sigma-Aldrich) or monosalicylate, (EGmSal, GC grade ≥98.0%, Sigma-Aldrich) was added into 10 g of DA-ODA:BADA PAA under an inert atmosphere and stirred continuously. Later, 0.1 g of *p*-toluenesulfonic acid monohydrate (ACS reagent, ≥98.5%, Sigma-Aldrich) was added as a mono-esterification catalyst, and the activation was conducted at 100 °C for 2 h. Once completed, the transesterification step was carried out after the solution casting on a glass plate, similarly to the discussed thermal imidization procedure between 70 and 300 °C. The procedure can also be found in other literature [15]. Moreover, crosslinking the 6FDA-ODA:BADA PAA was also conducted with the thermally labile unit, iron (III) acetylacetonate (FeAc, 97%, Sigma-Aldrich) where 0.2 g of the FeAc powder was added into 2.5 g of anhydrous DMF and sonicated for 2 h. A ca. 10 g of PAA was later added into the dispersed FeAc solution, making a diluted PAA solution of 8 wt.% polymer concentration with 2 wt.% of FeAc, to the polymer content. The new solution was then casted onto a glass plate and thermally imidized as the previous.

**Figure 4:**
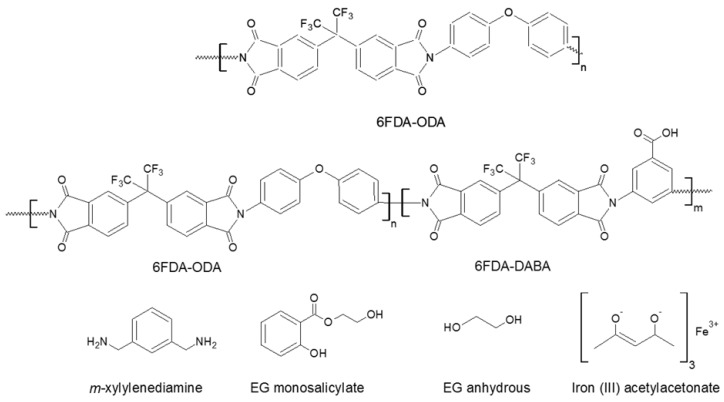
Chemical structures of 6FDA-ODA, 6FDA-ODA:DABA and several of their crosslinking agents in this study. The statistical copolymer of 6FDA-ODA:DABA is 1:X:Y where X = n/(n + m) and Y = m/(n + m).

The chemical structures of the polyimides and their crosslinking agents are represented in Figure 4.

The polymer’s functional groups were identified by a Perkin Elmer Fourier-transform infrared (FTIR) in the wavelength of 4000 cm^−1^ to 400 cm^−1^, at a resolution of 4 cm^−1^. It was also utilized to identify the anticipated atomic groups’ vibrations after the chemical crosslinking. A Hitachi 4700 scanning electron (SEM), equipped with a JEOL JSM-35C operated at 15 kV were utilized to image the membrane microstructures. The samples were placed on a carbon tape and coated with gold-palladium coating mixture for the analysis. A simultaneous thermogravimetric analysis (TGA) and differential scanning calorimetry (DSC) was carried out on a 7~15 mg sample using a Linseis STA 700LT at a constant heating rate of 10 °C min^−1^ up to 700 °C in N_2_. At the highest temperature, the combustion was conducted in the air for 40 min. The glass transition (T_g_) was determined by an inflection point of the specific heat curve obtained.

The extent of the membrane crosslinking was determined by calculating its gel content using Equation (1). A 0.4–0.5 g membrane was immersed in dimethylformamide (DMF, 99.8%, Sigma Aldrich) for 24 h [13]. The insoluble remains were filtered and dried in a vacuum oven at 200 °C for 24 h. M_0_ is defined as the membrane initial mass and M_1_ is its remaining mass.

(1) $$\mathrm{Gel}\;\mathrm{content},\;\%\;=\;\frac{M_{1}}{M_{0}}\;\times \;100\%$$

The fractional free volume, FFV of the membranes was calculated from the polymer specific volume, V = 1/ρ and occupied volume, V_0_ at −273 °C. It estimated at 1.288 times the Van der Waals volume (V_vdw_) [20]. The density measurement was conducted using a pycnometer (Picnomatic Thermo Fisher Scientific, Massachusetts, MO, USA) at 20 ± 0.01 °C where a ca. 100 mg sample was placed in the analysis cell and degassed using a series of pressurization He cycles at 2–20 bar. FFV is calculated as follows:

(2) $$\mathrm{Fractional}\;\mathrm{free}\;\mathrm{volume},\;\mathrm{FFV}=\frac{{V\;-\;V}_{0}}{V}{\;=\;1\;-\;\rho V}_{0}{;\;V}_{0}\;=\;{1.288\;\times \;V}_{\mathrm{vdw}}$$

The flat sheet membranes (ca. 25 mm in diameter) were tested using a steady-state apparatus as previously published [21], using the Wicke-Kallenbach method with an online Focus gas chromatography (GC). The GC is equipped with a flame ionization detector (FID) and a methanizer. An equimolar mixture of methane (>99.7%, 20 mL min^−1^, Linde, Munich, Germany) and carbon dioxide (>99.9%, 20 mL min^−1^, SIAD, Bergamo, Italy) was used as feed gas at 5 bar and 25 °C, with helium (99.999%, 5 mL min^−1^, SIAD) as a sweep gas. The permeability of the two gasses was determined by Equation (3), where y_CO2_ is CO_2_ molar fraction in the permeate and x_CO2_ in the feed gas. F^s^ is the calibrated sweep gas volumetric flow in cm^3^ (STP) s^−1^, l is membrane thickness in cm, P is the pressure in cm Hg, and A is the effective membrane area in cm^2^. The permeability is reported in Barrer (1 Barrer = 10^−10^ cm^3^ (STP) cm cm^−2^ s^−1^ cm Hg^−1^).

(3) $$P_{\mathrm{CO}2}=\frac{y_{\mathrm{CO}2}\cdot F^{s}\cdot l}{A\left(x_{\mathrm{CO}2}\cdot P^{R}{\;-\;y}_{\mathrm{CO}2}\cdot P^{P}\right)},$$

Selectivity values were determined using Equation (4), where x_i_ and y_i_ are the molar fractions in the feed and permeate stream, respectively.

(4) $$\alpha_{\mathrm{CO}2/\mathrm{CH}4}\;=\;\frac{y_{\mathrm{CO}2/\mathrm{CH}4}}{x_{\mathrm{CO}2/\mathrm{CH}4}},$$

## 3. Results and Discussion

### 3.1. Membrane Characterizations

**Figure 5:**
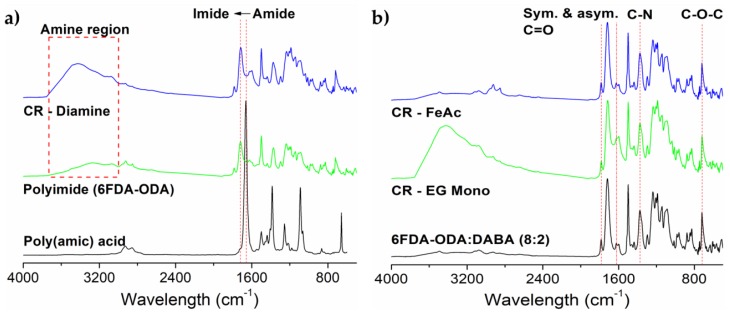
FTIR spectra of (**a**) neat 6FDA-ODA membrane (with the spectra of its poly(amic) acid, prior to the imidization and its crosslinked *m*-xylylene diamine membrane), and (**b**) neat 6FDA-ODA:DABA (8:2) membrane (with the spectra of its ethylene glycol (EG) and iron (III) acetylacetonate crosslinked membranes).

We conducted a FTIR analysis on the PAA and the produced neat membranes to determine the effectiveness of our imidization procedure. Figure 5a, which includes only 6FDA-ODA PAA and its imidized neat membrane for the discussion, indicates the disappearance of the PAA key functional group, amide –CONH– at 1656 cm^−1^ into imide, –NH– at 1720 cm^−1^. The amide into imide conversion also indicated by the disappearance of the carboxylic –OH at 2933 cm^−1^, due to PAA cyclodehydration and formation involving the amide’s nitrogen and the carboxylic acid’s oxygen to form an imide ring. This proves that the imidization procedure is sufficient to produce a polyimide. Other main imide peaks are defined as the symmetric C–N stretching at 1373 cm^−1^, both asymmetric C–O stretching at 1621 cm^−1^ and 1783 cm^−1^ and the ether –C–O–C– in ODA diamine moieties at 717 cm^−1^ (see Figure 5b) [22].

The analyses were also conducted on the crosslinked membranes to identify the presence of additional ‘bridging structures’ in the polymer matrix. As for the amine crosslinking, Figure 5a shows the spectra for *m*-xylylene diamine-crosslinked 6FDA-ODA and confirms the presence of additional amines when compared to the neat membrane in the amine region, marked in the red box. Similarly to the ethylene glycol crosslinking of 6FDA-ODA:DABA with EG monosalicylate (see Figure 5b), the broad convoluted peak between 3010 and 3750 cm^−1^ is attributed to several carboxylic –OH in the crosslinker.

**Figure 6:**
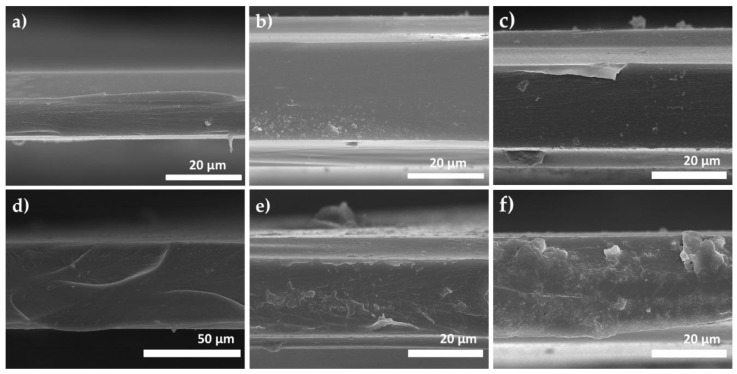
Cross-section SEM images of (**a**) neat 6FDA-ODA and its crosslinked membranes with (**b**) *m*-xylylene diamine and (**c**) *n*-ethylamine, (**d**) neat 6FDA-ODA:DABA (8:2) and its crosslinked membrane with (**e**) ethylene glycol monosalicylate and (**f**) iron (III) acetylacetonate.

Most importantly, we need to prove that the FeAc-crosslinked membrane preserved its backbone integrity after thermal annealing procedure to remove the acetylacetonate group as the procedure possesses a risk of polymer backbone degradation [17]. As shown in the FeAc-crosslinked sample’s spectra in Figure 5b, the integrity of 6FDA-ODA:DABA’s backbone is maintained and indicated by the presence of its symmetry and asymmetry C=O stretching at 1783 cm^−1^ and 1621 cm^−1^, –C–N– and –C–O–C– stretching at 1373 cm^−1^ and 717 cm^−1^, respectively. The microstructure of the flat sheet membranes was imaged by SEM (see Figure 6). The images show the membranes in the thickness range of 30–60 μm, are highly dense and defect-free, with no cracking or micro-void formation. Furthermore, the thicker flat sheet membranes required a more extended stabilization period in the permeation test, as it needs a longer time for the permeating gasses to saturate the polymer voids and to reach the permeation steady-state [23].

DSC measurements (Table 1) show the synthesized 6FDA-ODA transitioned to a rubbery polymer at 309 °C (glass transition temperature, T_g_), closed to the reported data at 294–303 °C [24,25], whereas the synthesized 6FDA-ODA:DABA (8:2) revealed two T_g_ at 263 °C and 327 °C. As the 6FDA-ODA were crosslinked with a diamine, ethylamine, and butylamine, the T_g_ increases by 4 °C, 9 °C and 13 °C, respectively. Likewise, crosslinking of 6FDA-ODA:DABA also causes rigidification of the polymer chains, thus limiting their movement and increased the corresponding T_g_ values; CR EG mono, T_g_ = 316 °C and CR FeAc, T_g_ = 313 °C. The higher T_g_ recorded by EG crosslinking is contributed by the additional formation of hydrogen bond in the presence of multiple hydroxyl, –OH groups in the crosslinker. The membranes thermal stabilities were characterized by TGA, and the corresponding decomposition temperatures (T_d_) were determined by the lowest convolution points of the weight loss derivative (see Table 1). Crosslinked 6FDA-ODA membranes show an increase of between 14 and 20 °C from the neat membrane (T_d_ = 549 °C), meanwhile lower T_d_ increases were recorded for crosslinked 6FDA-ODA:DABA membranes by only 7–10 °C, compared to its respective neat membrane (T_d_ = 538 °C). As expected, T_d_ increases with crosslinking due to higher polymer packing as the crosslinking agents tighten the polymer structure, and also the possibility of hydrogen bonds to occur, leading to a stronger intermolecular reaction.

As for the FFV values, it is important to note that the calculation is conducted to the polymers’ van der Waal’s volumes and their respective solid densities. The neat 6FDA-ODA and 6FDA-ODA:DABA (8:2) showed FFV values of 0.174 and 0.148, respectively. The values are close to the other reported FFVs for the polymers [24,25] and in the lower range of most polymer membranes (FFV = 0.1–0.3 [26]). As anticipated, crosslinking of the polymers produce membranes with lower FFV values, 6.9–19.5% reduction by amine crosslinking of 6FDA-ODA and 4.7–5.4% reduction by EG and FeAc crosslinking. It is clear that a greater FFV reduction is recorded when the crosslinking agent is a short rigid compound (i.e., *n*-ethylamine and *n*-butylamine) or contains hydrogen bond donor/acceptor functional groups (i.e., ethylene glycol monosalicylate). A more bulky component such *m*-xylylene diamine showed a lower FFV reduction, owing to its large aromatic group which hinders compaction of the chain packing.

Please note that there is no data or discussion on 6FDA-ODA:DABA crosslinked with EG anhydrous, because of that we were unsuccessful in producing the self-standing film. It is believed to be due to over-crosslinking by the short-length and rigid EG, instigating a very high rigidity polymer chain and causing membrane brittleness and cracking.

### 3.2. Gas Transport Properties

The mixed gas permeation properties of 6FDA-ODA, 6FDA-ODA:DABA and their crosslinked membranes were determined using an equimolar CO_2_:CH_4_ feed mixture at a constant pressure of 5 bar, at 25 °C. Neat 6FDA-ODA displays separation performances of P_CO2_ = 43.8 ± 1.6 Barrer and α_CO2/CH4_ = 29.9 ± 1.2, are comparable, if not higher to the values presented in earlier publications in the range of P_CO2_ = 11–26 Barrer and α_CO2/CH4_ = 26–52, also tested with an equimolar CO_2_:CH_4_ binary mixture between 2 to 5 bar, at 25–35 °C [6,24,27]. Nik et al. [25], however, reported a higher selectivity of α_CO2/CH4_ = 41.7 ± 2.3 and much lower permeability of P_CO2_ = 14.4 ± 0.6 for neat 6FDA-ODA. This may be attributed to their higher annealing temperature (at 230 °C, twice higher than our annealing temperature). A higher annealing temperature usually produces a higher polymer chain packing and denser membranes with lower permeability values and higher separation factors. The observation has also been reported in P84 polyimide [28] and polyamide-polysulfone-poly(ethylene terephthalate) thin film composite membranes [29,30], with regards to the temperature variation during membrane post-treatment. Gas separation of small kinetic molecules (CO_2_, CH_4_) in the membrane is governed by a diffusion mechanism, and the diffusion is enhanced in a higher free volume membranes [22,31]. As previously discussed, the crosslinked 6FDA-ODA membranes show lower FFV values and the relationship is evidently presented by their gas separation performances (Table 2). The *m*-xylylenediamine crosslinked 6FDA-ODA shows 76% P_CO2_ reduction with almost 100% in CO_2_/CH_4_ selectivity enhancement. 6FDA-ODA crosslinking with trimethylamine and 1-butylamine, on the other hand, reduced the P_CO2_ by 84% and 80%, respectively. In terms of CO_2_/CH_4_ selectivity, the trimethylamine and 1-butylamine crosslinked membranes showed lesser improvements of only 25% and 43%, respectively. The difference may be attributed to their lower crosslinking degree produced by these single-amine cross-linkers, as compared to diamine, and well-correlated to their gel content data presented in the previous section. As crosslinking tightens the polymer chain and lowers the gas diffusivity, the improvement is also believed to be contributed to by the inter-molecular hydrogen bonding between the cross-linkers and 6FDA-ODA’s hydrogen bond donor/acceptor groups (–CF3, –C=O, –CN– and –C–O–C– [24]).

Interestingly, 6FDA-ODA:DABA crosslinked with only 2 wt.% FeAc produces the best performing membrane, with P_CO2_ = 47.2 ± 1.5 Barrer and α_CO2/CH4_ = 40.0 ± 3.2, translated into 29% and 126% improvement in P_CO2_ and CO_2_/CH_4_ selectivity to the neat membrane, respectively. With this finding, it is proven that ion Fe^3+^ acts as a crosslinker where it reduces polymer chain mobility, subsequently increasing the separation factor, as expected. Meanwhile, the degraded acetylacetonate acts as a micro-pore former; thus, the observed permeability enhancement. The CO_2_ permeability increment is also contributed by its higher solubility in the polymer matrix due to CO_2_ having a greater affinity towards Fe^3+^ rather than the molecule with no unbounded electron pair, CH_4_ [8,32,33]. The finding contradicts the reported performance of FeAc-crosslinked 6FDA-Dureen:DABA by Chua et al. [17], where the CO_2_/CH_4_ selectivity only showed ±5% reductions in all membranes with 2–10 wt.% FeAc. They, however, mentioned that the variation in selectivity might be attributed to differences in the physiochemical properties of the crosslinked membranes, due to the different amount of iron (III) ions and the degree of cross-linking reaction. Previously, they also presented a similar observation when crosslinking 6FDA-Dureen:DABA with several thermally saccharide labile units (glucose, sucrose, and raffinose) [34].

**Figure 7:**
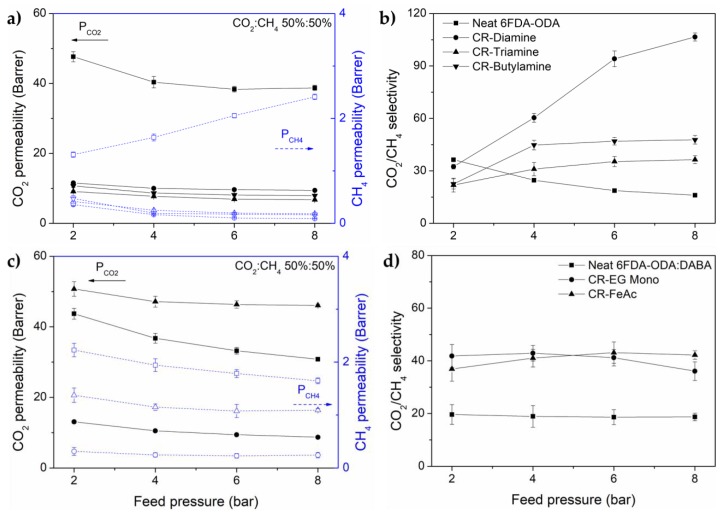
Gas permeability and CO_2_/CH_4_ selectivity of (**a**,**b**) 6FDA-ODA and (**c**,**d**) 6FDA-ODA:DABA and their respective crosslinked membranes, tested with an equimolar CO_2_/CH_4_ feed mixture at 2–8 bar. All measurement was conducted at 25 °C.

We investigated the gas separation performance of the membranes at a pressure ranging from 2 to 8 bar in a 50:50 vol.% CO_2_:CH_4_ feed mixture at 25 °C. The obtained mixed gas permeability and CO_2_/CH_4_ selectivity behavior as a function of pressure are shown in Figure 7. The CO_2_-induced plasticization pressure is defined to occur at the minimum observed in the CO_2_-permeability as a function of CO_2_-partial feed pressure [35]. In the case of neat 6FDA-ODA and its mixed gas separation, the permeation rate of all gasses is affected due to the polymer matrix swelling causing an increase in chain mobility by the high CO_2_ concentration. The effect is more pronounced in the least permeable gas (CH_4_), resulting in a decrease of CO_2_/CH_4_ selectivity, as a function of pressure (see Figure 7a,b). Nonetheless, the monotone decrease in CO_2_ permeability with increasing pressure indicates no substantial CO_2_-induced plasticization [36]; as for the other 6FDA-ODA crosslinked membranes, the typical dual-mode sorption and competitive effect [24,37] are observed where the gas permeability reduces continuously with the increasing pressure. The changes demonstrate the effectiveness of polymer crosslinking in suppressing the CO_2_-plasticization phenomenon in polymeric membranes.

Both neat 6FDA-ODA:DABA and EG mono-crosslinked membrane show competitive sorption effect and no polymer matrix swelling. The trends are similar to those of crosslinked 6FDA-ODA. Interesting, when to compared to the neat 6FDA-ODA, neat 6FDA-ODA:DABA shows no polymer swelling. This positive observation is attributed by DABA in the diamine moieties, where its carboxylic group (–COOH) acts as both hydrogen donor and acceptor to form an intra- and intermolecular interaction. The new bridged polymer possesses limited ability to rotate or move in the presence of a plasticization agent, thus deterring swelling of the polymer matrix [8,36]. The FeAc-crosslinked membrane presents a typical case and is similar to the crosslinked 6FDA-ODA membranes. The membrane also shows a CO_2_/CH_4_ selectivity increment of 14% when tested with a feed pressure between 2 to 8 bar. Nevertheless, the neat and EG mono-crosslinked membranes presented a slight CO_2_/CH_4_ selectivity reduction within its measurement error of ±4.2. The deviation is mainly contributed to by the very low CH_4_ permeation and its detection by our GC measurement.

As it is well-known, one of the key advantages of the membrane technology is its high adaptability to feed composition and process conditions [8]. Hence, further testing was conducted on the membranes to demonstrate its separation efficiency to different CO_2_ content (25–75 vol.%) in the binary feed mixture at 2 and 8 bar, at 25 °C.

**Figure 8:**
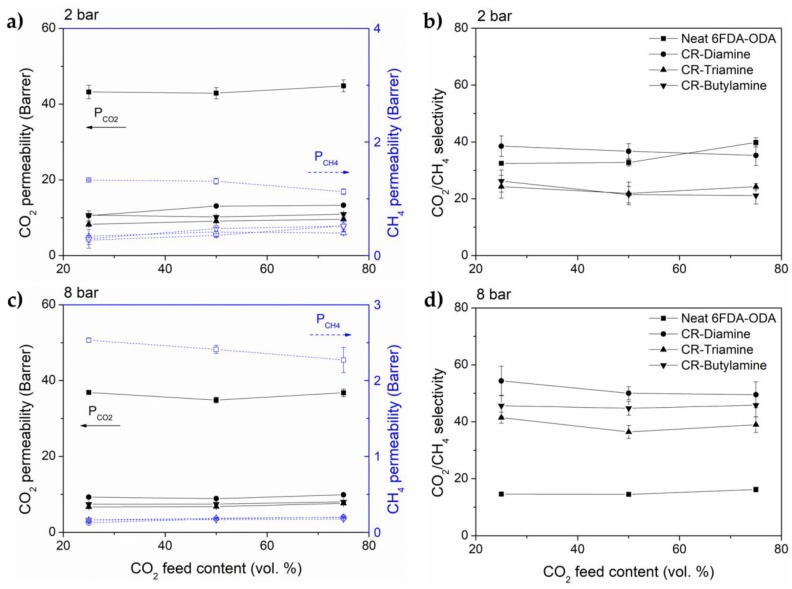

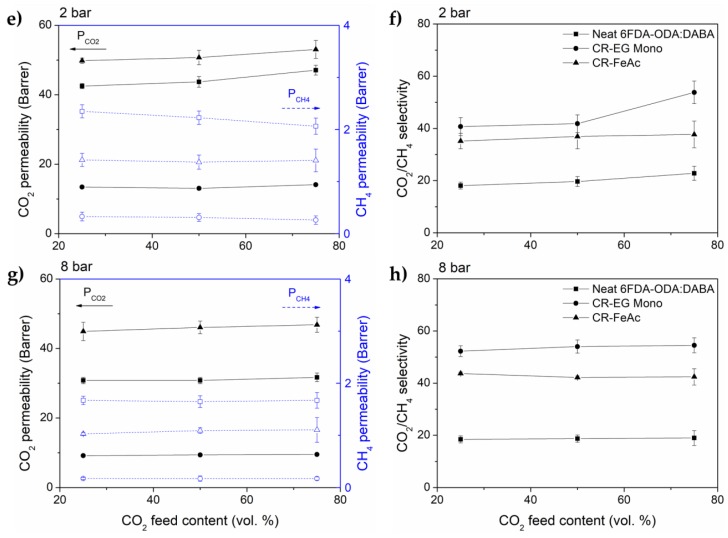
CO_2_ and CH_4_ permeability and CO_2_/CH_4_ selectivity of 6FDA-ODA and its crosslinked membranes at (**a**,**b**) 2 bar, (**c**,**d**) 8 bar; 6FDA-ODA:DABA and its crosslinked membranes at (**e**,**f**) 2 bar and (**g**,**h**) 8 bar, with 25–75 vol.% CO_2_ in the binary feed mixture with CH_4_. All measurement was conducted at 25 °C.

Commonly, CO_2_ permeability increases with increasing CO_2_ partial pressure in the feed gas, according to competitive sorption behavior where the higher CO_2_ partial pressure affects its diffusivity and solubility in the polymer matrix, and conversely decreases the least permeable gas permeability (in this case CH_4_) [8,38]. Thus, the selectivity improvement will be observed. At high pressure, CO_2_ permeability reduced with increasing CO_2_ partial pressure; this is believed to be more related to gradual saturation of the permeating gases inside the polymer permanent voids, affecting the overall mobility rather than the competitive sorption [39]. All of our neat samples follow predicted behavior at both feed pressures (see Figure 8). The CO_2_ partial pressure competitive sorption relationship is believed to more prominent at low pressures, as observed by Ahmad et al. [8] in 6FDA-DAM Zr-MOF (feed pressure at 2 bar) and Cakal et al. [40] in PES/SAPO-34/2-hydroxyl 5-methyl aniline (feed pressure 3 bar) mixed matrix membranes (MMMs). At 2 bar, neat 6FDA-ODA shows CO_2_ permeability increment by only 4% while CH_4_ permeability reduces by 15%, resulting in a 23% increment of CO_2_/CH_4_ selectivity. At the same pressure, neat 6FDA-ODA:DABA shows a higher increment of CO_2_ permeability by 11% and a bigger reduction in CH_4_ permeability by 12%. The difference here is again thought to be contributed to by the bulky aromatic DABA component in the diamine moieties. CO_2_/CH_4_ selectivity on the other hand increases by 26%. At the higher pressure of 8 bar, similar behavior was observed at lower permeability reductions (5–10% for 6FDA-ODA; 1–4% for 6FDA-ODA:DABA) and lower CO_2_/CH_4_ selectivity improvements (by only 11% for 6FDA-ODA; 3% for 6FDA-ODA:DABA) (see Figure 8). This indicated the feed pressure of 8 bar is simply not sufficient to give an observable gradual saturation effect in the polymer matrix. The behavior was recently presented by Ahmad et al. [8] in 6FDA-DAM Zr-MOF MMMs at a feed pressure of 40 bar. Overall, the similar gas permeability and selectivity trends were observed in all the crosslinked membranes.

### 3.3. Performance Benchmarking and Its Stability

**Figure 9:**
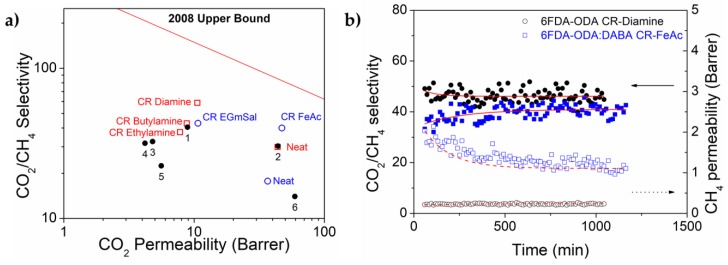
(**a**) The CO_2_/CH_4_ separation performance of neat 6FDA-ODA and its crosslinked membranes using (1) *m*-xylylene diamine; (2) *n*-ethylamine; (3) *n*-butylamine; neat 6FDA-ODA:DABA (8:2) and its crosslinked membranes with (i) EGmSal; (ii) FeAc, against the 2008 Robeson plot [4]. Also in comparison to several industrially relevant polymer membranes (numbered 1–6) for gas separation, as highlighted by Sanders et al. [41]; (**b**) The selectivity and CH_4_ permeability performances of 6FDA-ODA CR-diamine and 6FDA-ODA:DABA CR-FeAc, when tested with 50:50 CO_2_:CH_4_ feed mixture at 8 bar, at a constant temperature of 25 °C over time.

Figure 9a shows the performances of both 6FDA-based polyimide membranes and their respective crosslinked membranes with the CO_2_/CH_4_ Robeson upper bounds 2008 [4]. Indicated by the filled circles are industrially relevant polymers; (1) Matrimid^®^, (2) polyimide (PI), (3) cellulose acetate (CA), (4) tetrabromo polycarbonate (TBPC), (5) polysulfone (PSF), and (6) poly(2,6-dimethyl-1,4-phenylene oxide) (PPO), as highlighted in a review by Sanders et al. [41], for comparison. As depicted, the neat membranes reside well-below the upper bound, and the 6FDA-ODA membrane shows a good comparison to the commercial polyimide. All three amine crosslinking agents produced 6FDA-ODA membranes with higher CO_2_/CH_4_ selectivity at the expenses of CO_2_ permeability. The membranes also show better selectivity and CO_2_ permeability than CA, TBPC, and PSF. The size-sieving ability of the polymer was altered at a different rate depending on the crosslinking structures, their ‘bridge’ rigidity and also their crosslinking degree. A similar trend was displayed by 6FDA-ODA:DABA crosslinking with EG monosalicylate. Positively, FeAc-crosslinked 6FDA-ODA:DABA shows the ideal improvement where both selectivity and permeability were increased towards the upper bound, and performed superiorly to the commercialized polymers. The ‘residual’ Fe^3+^ ions upon thermal annealing also increase CO_2_ sorption solubility and thus the observed CO_2_ permeation enhancement. The best performing crosslinked membrane from each polymer—diamine-crosslinked 6FDA-ODA and FeAc-crosslinked 6FDA-ODA:DABA—were subjected to a durability test with 50:50 vol.% CO_2_:CH_4_ at the highest feed pressure of 8 bar and 25 °C. Their CO_2_/CH_4_ selectivity stabilities are presented in Figure 9b. Both samples demonstrated high selectivity stability at the tested condition, also observable in the steady permeation without any increment of the lower permeable component, CH_4_. The stable CH_4_ permeability increment proves that the membranes did not swell or plasticize in the presence of high CO_2_ content in the feed mixture over the test duration. However, due to the limitation of our permeation system, with a maximum safe operating pressure of only 8 bar, constant temperature operation and to further examine the stability of crosslinked membranes, we would like to suggest the following: (1) separation investigation at higher pressure and temperature, preferably simulating of an actual natural processing conditions (up to 30–60 bar, and 50–75 °C); (2) the separation stability in the presence of heavier hydrocarbons (C_2_–C_5_).

## 4. Conclusions

The chemical crosslinking of 6FDA-ODA with three types of amine were successful, and *m*-xylylene diamine was proven to be the most effective to increase the CO_2_/CH_4_ selectivity. Crosslinking of 6FDA-ODA:DABA with ethylene glycols (EGmSal; EGAn) and FeAc, on the other hand, demonstrated that the use of highly rigid and shorter component (EGAn) as the crosslinking agent, caused membrane brittleness and cracking. Nonetheless, the use of FeAc revealed that the compound could be the crosslinking agent of choice as it produced ideal separation enhancement, at only 2 wt.% addition. A further investigation for the FeAc optimum loading in 6FDA-ODA:DABA is believed to produce membranes with closer performance to or even surpassing the 2008 Robeson upper bound. This work demonstrated that chemical crosslinking, which is an easier and cheaper option, can produce the highly needed improvement and could be beneficial in the membrane optimization activities.

## Figures and Tables

**Table 1:** Physical properties of neat 6FDA-ODA, neat 6FDA-ODA:DABA (8:2) and their respective crosslinked membranes. FFV is calculated from the reciprocal density values, measured at 20 °C with pressurized He cycles between 2 and 20 bar.

Membranes	T_d_ (°C) ^a^	T_g_ (°C)	Density (g cm^−3^)	FFV ^b^
Neat 6FDA-ODA				
[24]	545	303	1.435	0.161
[25]	536	294	1.455	0.169
This study	549	309	1.413	0.174
CR Diamine	563	313	1.434	0.162
CR Ethylamine	567	318	1.451	0.152
CR Butylamine	569	322	1.471	0.140
Neat 6FDA-ODA:DABA (8:2)				
This study	538	263/327	1.366	0.148
CR EG Mono	545	316	1.379	0.140
CR FeAc	548	313	1.378	0.141

^a^ T_d_, σ ≤ 5% and T_g_, σ ≤ 8%, calculated from several independent measurements; ^b^ T_d_ is determined by the lowest inflection point of the TGA curve.

**Table 2:** CO_2_ and CH_4_ permeabilities and CO_2_/CH_4_ selectivity of the neat 6FDA-ODA and 6FDA-ODA:DABA and their crosslinked membranes, measured at 25 °C, feed pressure at 4 bar with an equimolar binary mixture of CO_2_ and CH_4_.

**Membranes**	**Neat 6FDA-ODA**	**CR-Diamine**	**CR-Triamine**	**CR-Butylamine**
Permeability (Barrer)				
CO_2_	43.8 ± 1.6	10.6 ± 0.2	7.8 ± 0.4	8.8 ± 0.3
CH_4_	1.5 ± 0.1	0.2 ± 0.0 *	0.2 ± 0.0 *	0.2 ± 0.0 *
Selectivity, α_CO2/CH4_	29.9 ± 1.2	58.8 ± 2.6	37.5 ± 3.8	42.9 ± 2.7
**Membranes**	**Neat 6FDA-ODA:DABA**		**CR-EG Mono**	**CR-FeAc**
Permeability (Barrer)				
CO_2_	36.7 ± 1.4		10.7 ± 0.3	47.2 ± 1.5
CH_4_	2.1 ± 0.1		0.2 ± 0.0 *	1.2 ± 0.1
Selectivity, α_CO2/CH4_	17.7 ± 4.1		43.0 ± 3.4	40.0 ± 3.2

* The relative error is in between ±0.01 and 0.04.
